# Crystal structures of the potassium and rubidium salts of (3,5-di­chloro­phen­oxy)acetic acid: two isotypic coordination polymers

**DOI:** 10.1107/S2056989015016722

**Published:** 2015-09-17

**Authors:** Graham Smith

**Affiliations:** a Science and Engineering Faculty, Queensland University of Technology, GPO Box 2434, Brisbane, Queensland 4001, Australia

**Keywords:** crystal structure, coordination polymers, (3,5-di­chloro­phen­oxy)acetic acid, 3,5-D, potassium and rubidium salts, hydrogen bonding

## Abstract

The two-dimensional coordination polymeric structures of the hydrated potassium and rubidium salts of (3,5-di­chloro­phen­oxy)acetic acid (3,5-D), namely, poly[μ-aqua-bis­[μ_3_-2-(3,5-di­chloro­phen­oxy)acetato]­dipotassium], [K_2_(C_8_H_5_Cl_2_O_3_)_2_(H_2_O)]_*n*_, and poly[μ-aqua-bis­[μ_3_-2-(3,5-di­chloro­phen­oxy)acetato]­dirubidium], [Rb_2_(C_8_H_5_Cl_2_O_3_)_2_(H_2_O)]_*n*_, respectively, have been determined and are described. The two compounds are isotypic and the polymeric structure is based on centrosymmetric dinuclear bridged complex units. The irregular six-coordination about the alkali cations comprises a bridging water mol­ecule lying on a twofold rotation axis, the phen­oxy O-atom donor and a triple bridging carboxyl­ate O atom of the oxo­acetate side chain of the 3,5-D ligand, and the second carb­oxy­ate O-atom donor also bridging. The K—O and Rb—O bond-length ranges are 2.7238 (15)–2.9459 (14) and 2.832 (2)–3.050 (2) Å, respectively, and the K⋯K and Rb⋯Rb separations in the dinuclear units are 4.0214 (7) and 4.1289 (6) Å, respectively. Within the layers which lie parallel to (100), the coordinating water mol­ecule forms an O—H⋯O hydrogen bond to the single bridging carboxyl­ate O atom.

## Abstract 2

The two compounds are isotypic and the two-dimensional polymeric structure is based on centrosymmetric dinuclear bridged complex units. Within the layers, which lie parallel to (100), the coordinating water mol­ecule forms an O—H⋯O hydrogen bond to the single bridging carboxyl­ate O atom.

## Chemical context   

The phen­oxy­acetic acids are a particularly useful series of compounds since certain members having specific ring-substituents have herbicidal activity, resulting in their being used commercially. Of these, the most common have been the chlorine-substituted analogues (2,4-di­chloro­phen­oxy)acetic acid (2,4-D), (2,4,5-tri­chloro­phen­oxy)acetic acid (2,4,5-T) and (4-chloro-2-methyl­phen­oxy)acetic acid (MCPA) (Zumdahl, 2010 ▸). As such, the active members have received considerable attention, particularly with respect to health aspects resulting from residual breakdown components after environmental exposure. Compounds formed from their reaction with a wide range of metals have provided a significant number of crystal structures, *e.g.* for 2,4-D, there are 60 examples of metal complexes, contained in the Cambridge Structural Database (CSD; Groom & Allen, 2014 ▸), *e.g.* with Ca^II^ (Song *et al.*, 2002 ▸) and with Zn^II^ (Kobylecka *et al.*, 2012 ▸).

Metal complex formation with the phen­oxy­acetic acids has been facilitated by their versatility as ligands, showing various inter­active modes with common metals including monodentate and bidentate-bridging coordinations involving the *O*_carbox­yl_, *O* ^1^_phen­oxy_ [(*O,O*)^1^] chelate inter­action, first reported for the monomeric copper(II) phen­oxy­acetate complex (Prout *et al.*, 1968 ▸) and also found in the potassium–2,4-D salt (Kennard *et al.*, 1983 ▸) as well as in the caesium complexes with 4-fluoro­phen­oxy­acetate and (4-chloro-2-meth­yl)phen­oxy­acetate (Smith, 2015*a* ▸). In the caesium complex-adduct with 2,4-D (Smith & Lynch, 2014 ▸), a tridentate chelate inter­action variant is found which includes, in addition to the *O,O* ^1^-chelate, a Cs—Cl bond to the *ortho*-Cl ring substituent of the ligand. Only occasional examples of the bidentate carboxyl­ate *O,O′*-chelate inter­action are found, *e.g.* with the previously mentioned caesium 4-fluoro­phen­oxy­acetate.

However, examples of structures of alkali metal salts of the phen­oxy­acetic acids are not common in the crystallographic literature, comprising, apart from the previously mentioned examples, the following: sodium phen­oxy­acetate hemihydrate (Prout *et al.*, 1971 ▸; Evans *et al.*, 2001 ▸), anhydrous caesium phen­oxy­acetate (Smith, 2014*a* ▸), the lithium, rubidium and caesium complexes of 2,4-D (Smith, 2015*a* ▸), caesium *o*-phenyl­ene­dioxydi­acetate dihydrate (Smith *et al.*, 1989 ▸) and the lithium salts of (2-chloro­phen­oxy)acetic acid (O’Reilly *et al.*, 1987 ▸), (2-carbamoylphen­oxy)acetic acid (Mak *et al.*, 1986 ▸) and (2-carb­oxy­phen­oxy)acetic acid (Smith *et al.*, 1986 ▸).
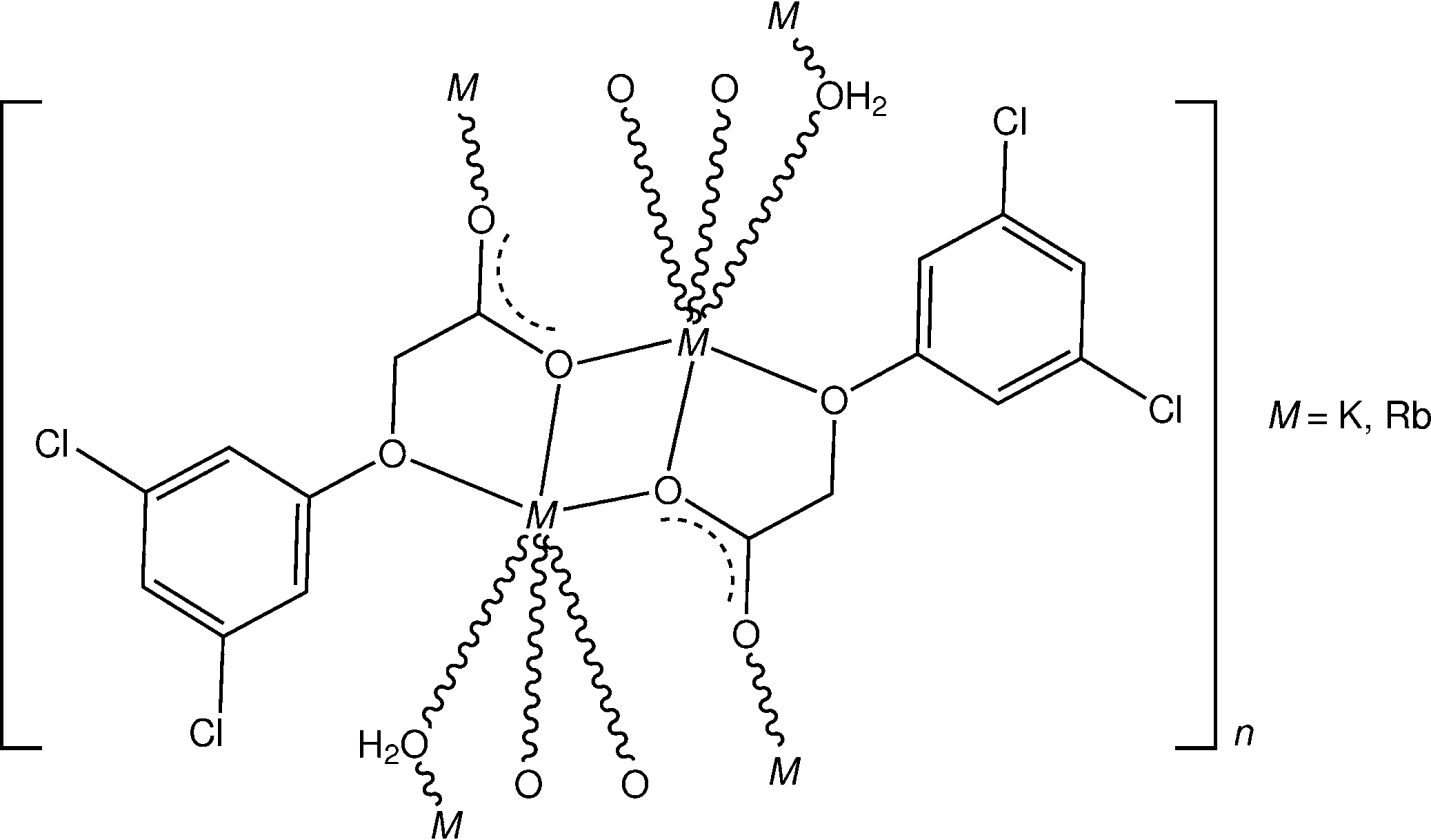


To investigate the nature of the coordination complex structures formed in the potassium and rubidium salts of the 2,4-D isomer, reactions of (3,5-di­chloro­phen­oxy)acetic acid (3,5-D) with K_2_CO_3_ and Rb_2_CO_3_ in aqueous ethanol were carried out, affording the isotypic polymeric title compounds [K_2_(C_8_H_5_Cl_2_O_3_)_2_(H_2_O)]_*n*_, (I), and [Rb_2_(C_8_H_5_Cl_2_O_3_)_2_(H_2_O)]_*n*_, (II), and the structures are reported herein.

## Structural commentary   

**Figure 1:**
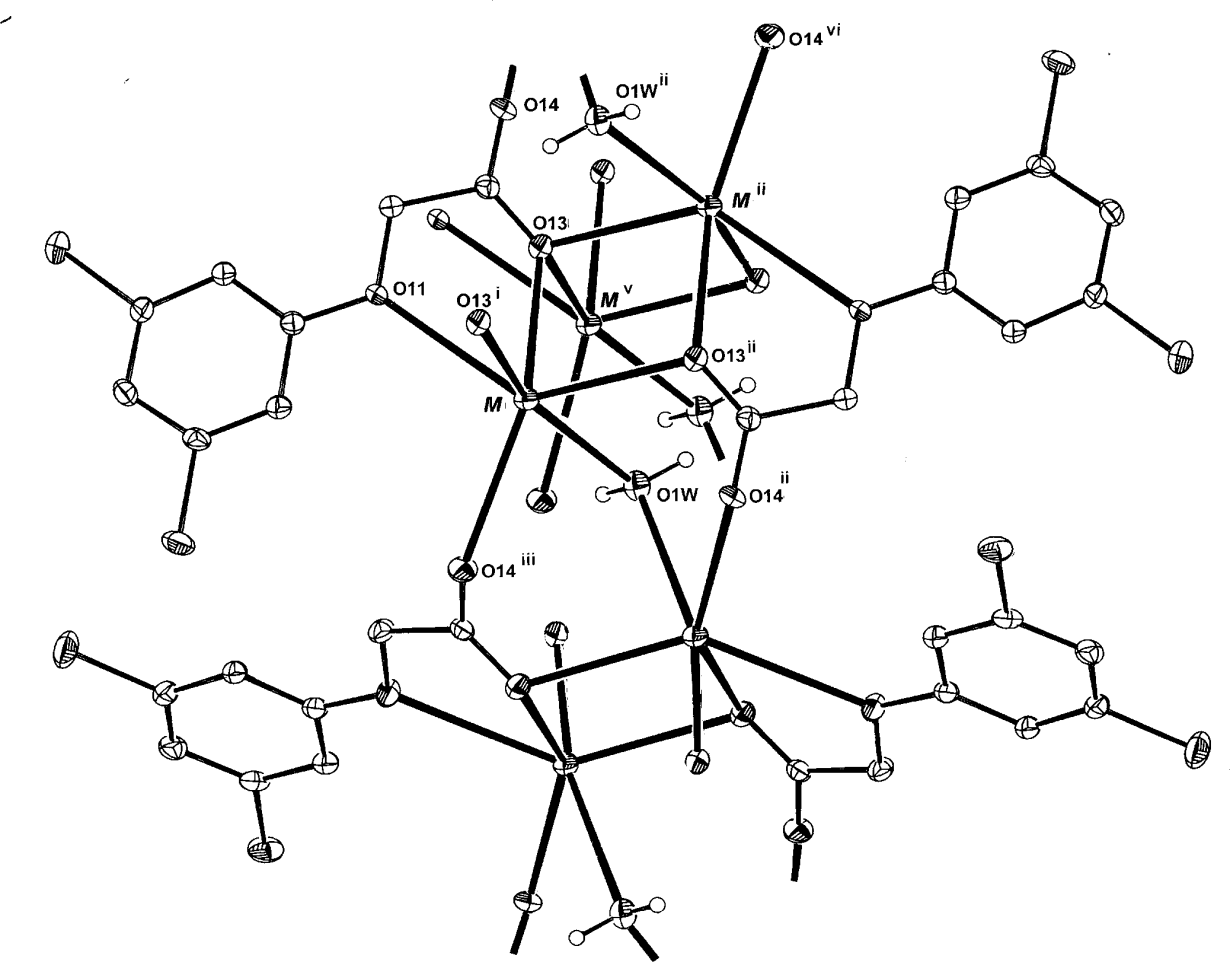
A view of the partially expanded polymeric extension of the structures of (I) and (II), shown with 30% probability ellipsoids (with data taken from the potassium structure). [See Table 1 ▸ for symmetry codes; additionally: (vi) *x* − 1, *y*, *z*; (vii) *x*, *y* − 1, *z*.]

**Figure 2:**
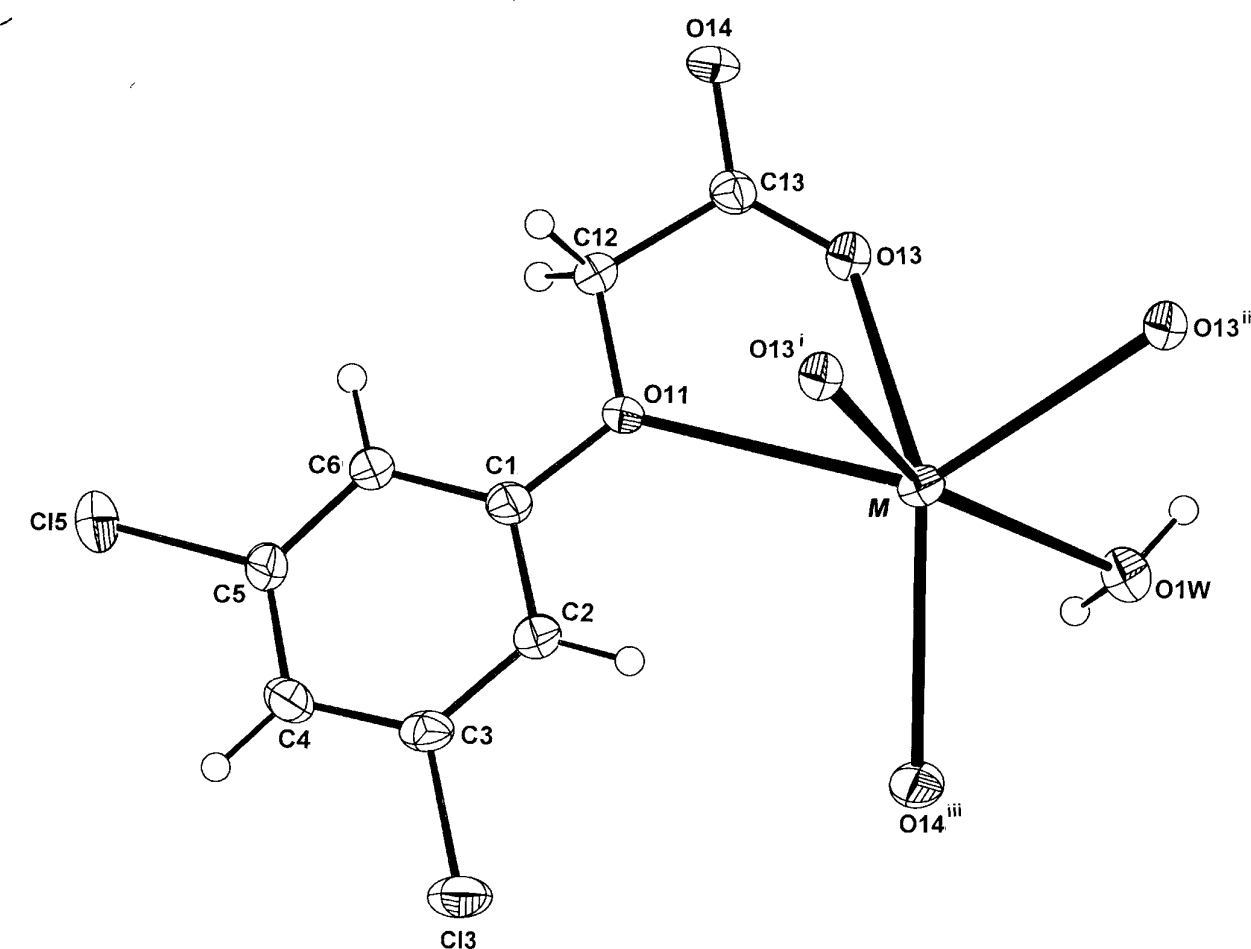
The mol­ecular configuration and atom-numbering scheme for the isomeric K and Rb complexes with 3,5-D [(I) and (II)], with displacement ellipsoids drawn at the 40% probability level (with data taken from the potassium structure). For symmetry codes, see Table 1 ▸.

The hydrated complexes (I) and (II) are isotypic and are described conjointly. Each comprises a centrosymmetric dinuclear repeating unit (Fig. 1 ▸) in which the irregular six-coordination about the K^+^ or Rb^+^ cations consists of a bidentate *O*_carboxyl­ate_ (O13), *O*_phen­oxy_ (O11) chelate inter­action (Fig. 2 ▸), three bridging carboxyl­ate (O13^i^, O13^ii^, O14^iii^; for symmetry codes, see Table 1 ▸) inter­actions and a single bridging water mol­ecule (O1*W*) lying on a twofold rotation axis. The comparative *M*—O bond length range for the two metals (Tables 1 ▸ and 2 ▸) is 2.7238 (15)–2.9459 (14) Å (K) and 2.832 (2)–3.050 (2) Å (Rb), for the two O-atom donors in the (*O*:*O* ^1^)-chelate inter­action (O13 and O11, respectively).

**Figure 3:**
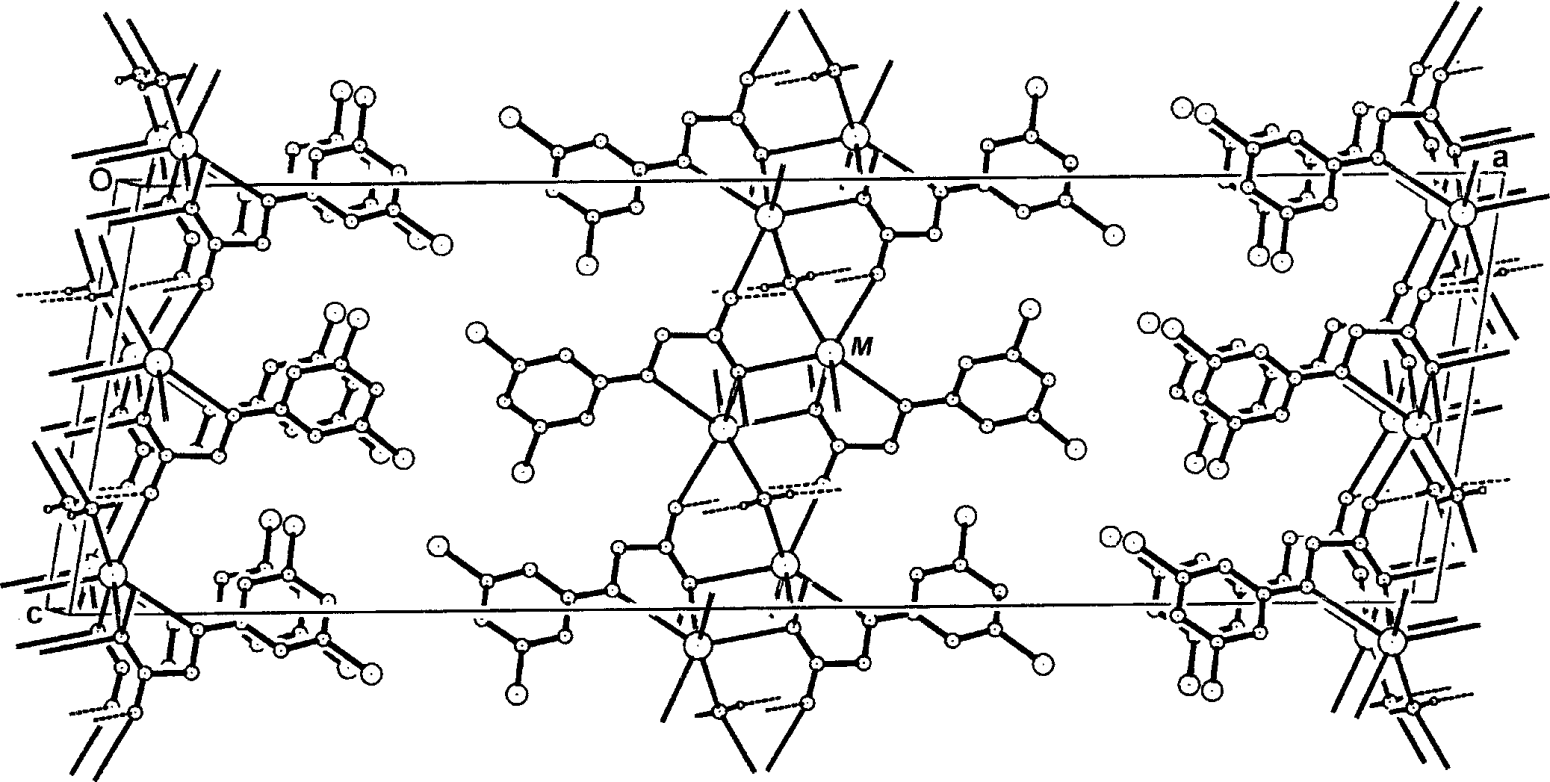
The packing of the layered structure of compounds (I) and (II) in the unit cell, viewed approximately along [010]. Non-associated H atoms have been omitted.

Two-dimensional coordination polymeric structures are generated, lying parallel to (100) (Fig. 3 ▸), in which the core sheet comprises the *M*—O complex network with the aromatic rings of the ligands peripherally located between the layers. Within the layers there are a number of short metal⋯metal contacts, the shortest being across an inversion centre [K⋯K^ii^ = 4.0214 (7) Å and Rb⋯Rb^ii^ = 4.1289 (6) Å], the longest being K⋯K^vi^ = 4.3327 (5) Å and Rb⋯Rb^vi^ = 4.5483 (5) Å [symmetry codes: (ii) −*x* + 1, −*y* + 1, −*z* + 1; (vi) −*x* + 1, *y*, −*z* + 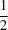]. No inter-ring π–π inter­actions are found in either (I) or (II), the minimum ring-centroid separations being 4.3327 (1) Å in (I) and 4.3302 (3) Å in (II), (the *b-*axis dimensions). The coordinating water mol­ecules on the twofold rotation axes are involved in intra-layer bridging O—H⋯O_carbox­yl_ hydrogen-bonding inter­actions (with O14 and O14^iv^) (Tables 3 ▸ and 4 ▸).

The 3,5-D anions in both (I) and (II) adopt the *anti­periplanar* conformation with the defining oxo­acetate side chain torsion angles C1—O11—C12—O13 of −171.55 (15) and −172.4 (2)° for (I), (II), respectively, that are similar to −172.4 (3)° in the ammonium salt (Smith, 2015*b* ▸). These values contrast with the value in the 2:1 3,5-D adduct with 4,4′-biphenyl [−71.6 (3)°] (*synclinal*) (Lynch *et al.*, 2003 ▸).

The present isotypic potassium and rubidium salts of (3,5-di­chloro­phen­oxy)acetic acid provide an example of isotypism which extends to the ammonium salt (Smith, 2015*b* ▸). Isotypism is also found in the analogous NH_4_^+^, K^+^ and Rb^+^ hemihydrate salts of isomeric 2,4-D (Table 5 ▸). It may also be possible that a similar series exists with MCPA for which the structure of only the ammonium hemihydrate salt (NH_4_^+^ MCPA^−^·0.5H_2_O) is known (Smith, 2014*b* ▸). It is of note that the sodium salts are not included in the sets, the structures for which are not known.

## Synthesis and crystallization   

Compounds (I) and (II) were synthesized by the addition of 0.5 mmol of K_2_CO_3_ (65 mg) [for (I)] or Rb_2_CO_3_ (115 mg) (for (II)] to a hot solution of (3,5-di­chloro­phen­oxy)acetic acid (3,5-D) (220 mg) in 10 ml of 50% (*v*/*v*) ethanol/water. After heating for 5 min, partial room temperature evaporation of the solutions gave in all two cases, colourless needles from which specimens were cleaved for the X-ray analyses.

## Refinement details   

Crystal data, data collection and structure refinement details for (I) and (II) are summarized in Table 6 ▸. Hydrogen atoms were placed in calculated positions [C—H_aromatic_ = 0.95 Å or C—H_methyl­ene_ = 0.99 Å] and were allowed to ride in the refinements, with *U*_iso_(H) = 1.2*U*_eq_(C). The water H-atom in both structures was located in a difference Fourier map and was allowed to ride in the refinements with an O—H distance restraint of 0.90±0.02 Å and with *U*_iso_(H) = 1.5*U*_eq_(O).

## Supplementary Material

Crystal structure: contains datablock(s) global, I, II. DOI: 10.1107/S2056989015016722/wm5206sup1.cif

Structure factors: contains datablock(s) I. DOI: 10.1107/S2056989015016722/wm5206Isup2.hkl

Structure factors: contains datablock(s) II. DOI: 10.1107/S2056989015016722/wm5206IIsup3.hkl

Supporting information file. DOI: 10.1107/S2056989015016722/wm5206Isup4.cml

Supporting information file. DOI: 10.1107/S2056989015016722/wm5206IIsup5.cml

CCDC references: 1422835, 1422834

Additional supporting information:  crystallographic information; 3D view; checkCIF report

## Figures and Tables

**Table 1:** Selected bond lengths (Å) for (I)

K1—O1*W*	2.7947 (15)	K1—O13^i^	2.7855 (15)
K1—O11	2.9459 (14)	K1—O13^ii^	2.7462 (13)
K1—O13	2.7238 (15)	K1—O14^iii^	2.7309 (16)

Symmetry codes: (i) 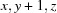; (ii) 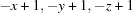; (iii) 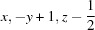.

**Table 2:** Selected bond lengths (Å) for (II)

Rb1—O1*W*	2.924 (2)	Rb1—O13^i^	2.874 (2)
Rb1—O11	3.050 (2)	Rb1—O13^ii^	2.894 (2)
Rb1—O13	2.832 (2)	Rb1—O14^iii^	2.842 (2)

Symmetry codes: (i) 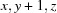; (ii) 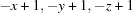; (iii) 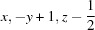.

**Table 3:** Hydrogen-bond geometry (Å, °) for (I)

*D*—H⋯*A*	*D*—H	H⋯*A*	*D*⋯*A*	*D*—H⋯*A*
O1*W*—H1*W*⋯O14^iv^	0.85 (2)	1.90 (2)	2.750 (2)	174 (2)

Symmetry code: (iv) 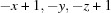.

**Table 4:** Hydrogen-bond geometry (Å, °) for (II)

*D*—H⋯*A*	*D*—H	H⋯*A*	*D*⋯*A*	*D*—H⋯*A*
O1*W*—H1*W*⋯O14^iv^	0.89 (3)	1.87 (3)	2.750 (3)	171 (5)

Symmetry code: (iv) 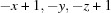.

**Table 5:** Comparative cell data (Å, °, Å^3^) for NH_4_^+^, K^+^ and Rb^+^ salts of (3,5-di­chloro­phen­oxy)acetic acid (3,5-D), (2,4-di­chloro­phen­oxy)acetic acid (2,4-D) and (4-chloro-2-methyl­phen­oxy)acetic acid (MCPA)

Cell parameters	NH_4_^+^3,5-D^−^·0.5H_2_O	K^+^3,5-D^−^·0.5H_2_O	Rb^+^3,5-D^−^·0.5H_2_O	NH_4_^+^2,4-D^−^·0.5H_2_O	K^+^2,4-D^−^·0.5H_2_O	Rb^+^2,4-D^−^·0.5H_2_O	NH_4_^+^MCPA^−^·0.5H_2_O
*a*	39.818 (3)	39.274 (2)	39.641 (3)	39.3338 (8)	36.80 (1)	37.254 (2)	38.0396 (9)
*b*	4.3340 (4)	4.3327 (3)	4.3302 (3)	4.3889 (9)	4.339 (1)	4.3589 (3)	4.456 (5)
*c*	12.7211 (8)	12.4234 (10)	12.8607 (8)	12.900 (3)	12.975 (7)	13.238 (1)	12.944 (5)
β (°)	98.098 (5)	99.363 (6)	98.404 (5)	103.83 (3)	102.03 (4)	103.231 (7)	104.575 (5)
*V*	2178.4 (5)	2085.8 (3)	2183.9 (3)	2074.7 (8)	2026 (2)	2092.6 (3)	2123 (3)
*Z*	8	8	8	8	8	8	8
Space group	*C*2/*c*	*C*2/*c*	*C*2/*c*	*C*2/*c*	*C*2/*c*	*C*2/*c*	*C*2/*c*
Reference	Smith (2015*b* ▸)	This work (I)	This work (II)	Liu *et al.* (2009 ▸)	Smith (2015*a* ▸)	Smith (2015*a* ▸)	Smith (2014*b* ▸)

**Table 6:** Experimental details

	(I)	(II)
Crystal data		
Chemical formula	[K_2_(C_8_H_5_Cl_2_O_3_)_2_(H_2_O)]	[Rb_2_(C_8_H_5_Cl_2_O_3_)_2_(H_2_O)]
*M*_r_	536.26	629.00
Crystal system, space group	Monoclinic, *C*2/*c*	Monoclinic, *C*2/*c*
Temperature (K)	200	200
*a*, *b*, *c* (Å)	39.274 (2), 4.3327 (3), 12.4234 (10)	39.641 (3), 4.3302 (3), 12.8607 (8)
β (°)	99.363 (6)	98.404 (5)
*V* (Å^3^)	2085.8 (3)	2183.9 (3)
*Z*	4	4
Radiation type	Mo *K*α	Mo *K*α
μ (mm^−1^)	1.00	5.01
Crystal size (mm)	0.45 × 0.12 × 0.04	0.40 × 0.12 × 0.04

Data collection		
Diffractometer	Oxford Diffraction Gemini-S CCD detector	Oxford Diffraction Gemini-S CCD detector
Absorption correction	Multi-scan (*CrysAlis PRO*; Agilent, 2013 ▸)	Multi-scan (*CrysAlis PRO*; Agilent, 2013 ▸)
*T*_min_, *T*_max_	0.774, 0.980	0.369, 0.980
No. of measured, independent and observed [*I* > 2σ(*I*)] reflections	6745, 2061, 1824	7520, 2152, 1910
*R*_int_	0.035	0.055
(sin θ/λ)_max_ (Å^−1^)	0.617	0.617

Refinement		
*R*[*F* ^2^ > 2σ(*F* ^2^)], *wR*(*F* ^2^), *S*	0.031, 0.076, 1.07	0.040, 0.095, 1.06
No. of reflections	2061	2152
No. of parameters	135	136
No. of restraints	1	1
H-atom treatment	H atoms treated by a mixture of independent and constrained refinement	H atoms treated by a mixture of independent and constrained refinement
Δρ_max_, Δρ_min_ (e Å^−3^)	0.27, −0.25	0.98, −1.00

Computer programs: *CrysAlis PRO* (Agilent, 2013 ▸), *SIR92* (Altomare *et al.*, 1993 ▸), *SHELXS97* and *SHELXL97* (Sheldrick, 2008 ▸) within *WinGX* (Farrugia, 2012 ▸), *PLATON* (Spek, 2009 ▸).

## supplementary crystallographic information

### (I) Poly[µ-aqua-bis[µ3-2-(3,5-dichlorophenoxy)acetato]dipotassium] Crystal data

**Table:**

[K_2_(C_8_H_5_Cl_2_O_3_)_2_(H_2_O)]	*F*(000) = 1080
*M**_r_* = 536.26	*D*_x_ = 1.708 Mg m^−^^3^
Monoclinic, *C*2/*c*	Mo *K*α radiation, λ = 0.71073 Å
Hall symbol: -C 2yc	Cell parameters from 2400 reflections
*a* = 39.274 (2) Å	θ = 4.2–28.6°
*b* = 4.3327 (3) Å	µ = 1.00 mm^−^^1^
*c* = 12.4234 (10) Å	*T* = 200 K
β = 99.363 (6)°	Flat prism, colourless
*V* = 2085.8 (3) Å^3^	0.45 × 0.12 × 0.04 mm
*Z* = 4	

### (I) Poly[µ-aqua-bis[µ3-2-(3,5-dichlorophenoxy)acetato]dipotassium] Data collection

**Table:**

Oxford Diffraction Gemini-S CCD-detector diffractometer	2061 independent reflections
Radiation source: Enhance (Mo) X-ray source	1824 reflections with *I* > 2σ(*I*)
Graphite monochromator	*R*_int_ = 0.035
Detector resolution: 16.077 pixels mm^-1^	θ_max_ = 26.0°, θ_min_ = 3.2°
ω scans	*h* = −48→47
Absorption correction: multi-scan (*CrysAlis PRO*; Agilent, 2013)	*k* = −5→5
*T*_min_ = 0.774, *T*_max_ = 0.980	*l* = −15→15
6745 measured reflections	

### (I) Poly[µ-aqua-bis[µ3-2-(3,5-dichlorophenoxy)acetato]dipotassium] Refinement

**Table:**

Refinement on *F*^2^	Primary atom site location: structure-invariant direct methods
Least-squares matrix: full	Secondary atom site location: difference Fourier map
*R*[*F*^2^ > 2σ(*F*^2^)] = 0.031	Hydrogen site location: inferred from neighbouring sites
*wR*(*F*^2^) = 0.076	H atoms treated by a mixture of independent and constrained refinement
*S* = 1.07	*w* = 1/[σ^2^(*F*_o_^2^) + (0.0337*P*)^2^ + 0.706*P*] where *P* = (*F*_o_^2^ + 2*F*_c_^2^)/3
2061 reflections	(Δ/σ)_max_ = 0.001
135 parameters	Δρ_max_ = 0.27 e Å^−^^3^
1 restraint	Δρ_min_ = −0.25 e Å^−^^3^

### (I) Poly[µ-aqua-bis[µ3-2-(3,5-dichlorophenoxy)acetato]dipotassium] Special details

**Table:**

Geometry. Bond distances, angles etc. have been calculated using the rounded fractional coordinates. All su's are estimated from the variances of the (full) variance-covariance matrix. The cell esds are taken into account in the estimation of distances, angles and torsion angles
Refinement. Refinement of F^2^ against ALL reflections. The weighted R-factor wR and goodness of fit S are based on F^2^, conventional R-factors R are based on F, with F set to zero for negative F^2^. The threshold expression of F^2^ > 2sigma(F^2^) is used only for calculating R-factors(gt) etc. and is not relevant to the choice of reflections for refinement. R-factors based on F^2^ are statistically about twice as large as those based on F, and R- factors based on ALL data will be even larger.

### (I) Poly[µ-aqua-bis[µ3-2-(3,5-dichlorophenoxy)acetato]dipotassium] Fractional atomic coordinates and isotropic or equivalent isotropic displacement parameters (Å^2^)

**Table:**

	*x*	*y*	*z*	*U*_iso_*/*U*_eq_	
K1	0.53071 (1)	0.71864 (10)	0.40994 (4)	0.0253 (1)	
Cl3	0.66484 (1)	1.12106 (12)	0.30636 (5)	0.0351 (2)	
Cl5	0.72749 (1)	0.44252 (15)	0.64380 (5)	0.0436 (2)	
O1W	0.50000	0.3066 (5)	0.25000	0.0301 (7)	
O11	0.59608 (3)	0.5041 (3)	0.53873 (12)	0.0279 (4)	
O13	0.53561 (3)	0.2277 (3)	0.54855 (12)	0.0279 (4)	
O14	0.55253 (4)	0.0910 (3)	0.72297 (12)	0.0317 (5)	
C1	0.62867 (5)	0.5876 (4)	0.52303 (17)	0.0226 (6)	
C2	0.63030 (5)	0.7874 (4)	0.43626 (17)	0.0243 (6)	
C3	0.66234 (5)	0.8758 (4)	0.41548 (17)	0.0250 (6)	
C4	0.69289 (5)	0.7753 (5)	0.47741 (18)	0.0286 (6)	
C5	0.69014 (5)	0.5791 (5)	0.56273 (18)	0.0268 (6)	
C6	0.65879 (5)	0.4817 (5)	0.58735 (17)	0.0242 (6)	
C12	0.59359 (5)	0.3273 (5)	0.63485 (17)	0.0276 (6)	
C13	0.55716 (5)	0.2100 (4)	0.63421 (17)	0.0228 (6)	
H1W	0.4837 (5)	0.189 (5)	0.263 (2)	0.0340*	
H2	0.60980	0.86110	0.39240	0.0290*	
H4	0.71470	0.83880	0.46170	0.0340*	
H6	0.65780	0.34570	0.64670	0.0290*	
H121	0.60060	0.45750	0.70020	0.0330*	
H122	0.60960	0.14980	0.63920	0.0330*	

### (I) Poly[µ-aqua-bis[µ3-2-(3,5-dichlorophenoxy)acetato]dipotassium] Atomic displacement parameters (Å^2^)

**Table:**

	*U*^11^	*U*^22^	*U*^33^	*U*^12^	*U*^13^	*U*^23^
K1	0.0223 (2)	0.0305 (2)	0.0223 (3)	−0.0004 (2)	0.0016 (2)	−0.0001 (2)
Cl3	0.0440 (3)	0.0342 (3)	0.0282 (3)	−0.0098 (2)	0.0095 (3)	0.0029 (2)
Cl5	0.0188 (3)	0.0630 (4)	0.0457 (4)	−0.0017 (3)	−0.0046 (2)	0.0085 (3)
O1W	0.0230 (11)	0.0293 (11)	0.0381 (14)	0.0000	0.0051 (10)	0.0000
O11	0.0163 (7)	0.0415 (8)	0.0251 (8)	−0.0026 (6)	0.0011 (6)	0.0101 (7)
O13	0.0197 (7)	0.0353 (8)	0.0266 (8)	−0.0036 (6)	−0.0028 (6)	−0.0003 (7)
O14	0.0293 (8)	0.0418 (9)	0.0251 (9)	−0.0062 (7)	0.0075 (7)	0.0028 (7)
C1	0.0185 (10)	0.0278 (10)	0.0214 (11)	−0.0023 (8)	0.0029 (8)	−0.0037 (9)
C2	0.0228 (10)	0.0267 (10)	0.0226 (11)	−0.0002 (8)	0.0015 (8)	−0.0016 (9)
C3	0.0302 (11)	0.0243 (10)	0.0211 (11)	−0.0049 (9)	0.0061 (9)	−0.0034 (9)
C4	0.0222 (10)	0.0348 (11)	0.0297 (12)	−0.0077 (9)	0.0070 (9)	−0.0070 (10)
C5	0.0180 (10)	0.0338 (11)	0.0266 (12)	−0.0019 (8)	−0.0023 (8)	−0.0039 (9)
C6	0.0206 (10)	0.0303 (10)	0.0213 (11)	−0.0027 (8)	0.0021 (8)	−0.0005 (9)
C12	0.0232 (11)	0.0384 (11)	0.0200 (11)	−0.0054 (9)	0.0002 (9)	0.0063 (9)
C13	0.0196 (10)	0.0233 (9)	0.0256 (12)	0.0003 (8)	0.0039 (9)	−0.0039 (9)

### (I) Poly[µ-aqua-bis[µ3-2-(3,5-dichlorophenoxy)acetato]dipotassium] Geometric parameters (Å, º)

**Table:**

K1—O1W	2.7947 (15)	O1W—H1W^iv^	0.85 (2)
K1—O11	2.9459 (14)	C1—C6	1.393 (3)
K1—O13	2.7238 (15)	C1—C2	1.392 (3)
K1—O13^i^	2.7855 (15)	C2—C3	1.379 (3)
K1—O13^ii^	2.7462 (13)	C3—C4	1.386 (3)
K1—O14^iii^	2.7309 (16)	C4—C5	1.377 (3)
Cl3—C3	1.738 (2)	C5—C6	1.382 (3)
Cl5—C5	1.742 (2)	C12—C13	1.517 (3)
O11—C1	1.374 (2)	C2—H2	0.9500
O11—C12	1.435 (3)	C4—H4	0.9500
O13—C13	1.250 (2)	C6—H6	0.9500
O14—C13	1.257 (2)	C12—H121	0.9900
O1W—H1W	0.85 (2)	C12—H122	0.9900
			
O1W—K1—O11	114.95 (4)	H1W—O1W—H1W^iv^	107 (2)
O1W—K1—O13	85.90 (5)	K1^iv^—O1W—H1W^iv^	119.8 (16)
O1W—K1—O13^i^	157.48 (4)	O11—C1—C2	115.78 (17)
O1W—K1—O13^ii^	82.81 (3)	O11—C1—C6	123.74 (18)
O1W—K1—O14^iii^	75.35 (4)	C2—C1—C6	120.48 (18)
O11—K1—O13	56.26 (4)	C1—C2—C3	118.42 (18)
O11—K1—O13^i^	87.00 (4)	C2—C3—C4	122.86 (19)
O11—K1—O13^ii^	133.96 (4)	Cl3—C3—C4	118.13 (15)
O11—K1—O14^iii^	101.01 (4)	Cl3—C3—C2	119.01 (15)
O13—K1—O13^i^	103.70 (4)	C3—C4—C5	116.88 (18)
O13—K1—O13^ii^	85.35 (4)	Cl5—C5—C4	119.39 (16)
O13—K1—O14^iii^	140.71 (4)	C4—C5—C6	122.91 (19)
O13^i^—K1—O13^ii^	77.83 (4)	Cl5—C5—C6	117.71 (17)
O13^i^—K1—O14^iii^	106.68 (4)	C1—C6—C5	118.45 (19)
O13^ii^—K1—O14^iii^	124.93 (5)	O11—C12—C13	111.48 (16)
K1—O1W—K1^iv^	100.60 (7)	O13—C13—C12	119.43 (18)
K1—O11—C1	126.11 (11)	O14—C13—C12	113.81 (18)
K1—O11—C12	116.68 (10)	O13—C13—O14	126.70 (18)
C1—O11—C12	116.72 (15)	C1—C2—H2	121.00
K1—O13—C13	123.69 (11)	C3—C2—H2	121.00
K1—O13—K1^v^	103.70 (5)	C3—C4—H4	122.00
K1—O13—K1^ii^	94.65 (4)	C5—C4—H4	122.00
K1^v^—O13—C13	116.55 (11)	C1—C6—H6	121.00
K1^ii^—O13—C13	112.14 (12)	C5—C6—H6	121.00
K1^v^—O13—K1^ii^	102.18 (4)	O11—C12—H121	109.00
K1^vi^—O14—C13	137.09 (12)	O11—C12—H122	109.00
K1^iv^—O1W—H1W	105.4 (15)	C13—C12—H121	109.00
K1—O1W—H1W	119.8 (16)	C13—C12—H122	109.00
K1—O1W—H1W^iv^	105.4 (15)	H121—C12—H122	108.00
			
O11—K1—O1W—K1^iv^	−146.99 (3)	O13—K1—O13^ii^—K1^ii^	−0.02 (5)
O13—K1—O1W—K1^iv^	163.37 (3)	O13—K1—O13^ii^—C13^ii^	−129.34 (12)
O1W—K1—O11—C1	99.66 (13)	O11—K1—O14^iii^—C13^iii^	87.4 (2)
O1W—K1—O11—C12	−88.68 (13)	O13—K1—O14^iii^—C13^iii^	38.4 (2)
O13—K1—O11—C1	165.74 (15)	K1—O11—C1—C2	−1.4 (2)
O13—K1—O11—C12	−22.60 (12)	K1—O11—C1—C6	179.21 (14)
O13^i^—K1—O11—C1	−85.59 (14)	C12—O11—C1—C2	−173.08 (17)
O13^i^—K1—O11—C12	86.08 (12)	C12—O11—C1—C6	7.6 (3)
O13^ii^—K1—O11—C1	−155.47 (13)	K1—O11—C12—C13	15.98 (19)
O13^ii^—K1—O11—C12	16.20 (14)	C1—O11—C12—C13	−171.55 (15)
O14^iii^—K1—O11—C1	20.83 (14)	K1—O13—C13—O14	143.75 (15)
O14^iii^—K1—O11—C12	−167.51 (12)	K1—O13—C13—C12	−39.2 (2)
O1W—K1—O13—C13	156.32 (14)	K1^v^—O13—C13—O14	−85.6 (2)
O1W—K1—O13—K1^v^	20.65 (4)	K1^v^—O13—C13—C12	91.41 (17)
O1W—K1—O13—K1^ii^	−83.10 (4)	K1^ii^—O13—C13—O14	31.6 (2)
O11—K1—O13—C13	32.52 (14)	K1^ii^—O13—C13—C12	−151.35 (14)
O11—K1—O13—K1^v^	−103.16 (5)	K1^vi^—O14—C13—O13	−90.6 (2)
O11—K1—O13—K1^ii^	153.10 (6)	K1^vi^—O14—C13—C12	92.3 (2)
O13^i^—K1—O13—C13	−44.32 (15)	O11—C1—C2—C3	−179.06 (16)
O13^i^—K1—O13—K1^v^	179.98 (9)	C6—C1—C2—C3	0.3 (3)
O13^i^—K1—O13—K1^ii^	76.26 (5)	O11—C1—C6—C5	179.20 (18)
O13^ii^—K1—O13—C13	−120.58 (14)	C2—C1—C6—C5	−0.1 (3)
O13^ii^—K1—O13—K1^v^	103.75 (5)	C1—C2—C3—Cl3	179.16 (14)
O13^ii^—K1—O13—K1^ii^	0.02 (8)	C1—C2—C3—C4	−0.3 (3)
O14^iii^—K1—O13—C13	95.53 (16)	Cl3—C3—C4—C5	−179.45 (16)
O14^iii^—K1—O13—K1^v^	−40.15 (8)	C2—C3—C4—C5	0.0 (3)
O14^iii^—K1—O13—K1^ii^	−143.89 (6)	C3—C4—C5—Cl5	179.79 (16)
O11—K1—O13^i^—K1^i^	125.82 (4)	C3—C4—C5—C6	0.2 (3)
O11—K1—O13^i^—C13^i^	−13.64 (13)	Cl5—C5—C6—C1	−179.71 (16)
O13—K1—O13^i^—K1^i^	180.00 (4)	C4—C5—C6—C1	−0.2 (3)
O13—K1—O13^i^—C13^i^	40.53 (13)	O11—C12—C13—O13	12.0 (2)
O11—K1—O13^ii^—K1^ii^	−31.51 (7)	O11—C12—C13—O14	−170.65 (16)
O11—K1—O13^ii^—C13^ii^	−160.85 (11)		

Symmetry codes: (i) *x*, *y*+1, *z*; (ii) −*x*+1, −*y*+1, −*z*+1; (iii) *x*, −*y*+1, *z*−1/2; (iv) −*x*+1, *y*, −*z*+1/2; (v) *x*, *y*−1, *z*; (vi) *x*, −*y*+1, *z*+1/2.

### (I) Poly[µ-aqua-bis[µ3-2-(3,5-dichlorophenoxy)acetato]dipotassium] Hydrogen-bond geometry (Å, º)

**Table:**

*D*—H···*A*	*D*—H	H···*A*	*D*···*A*	*D*—H···*A*
O1*W*—H1*W*···O14^vii^	0.85 (2)	1.90 (2)	2.750 (2)	174 (2)

Symmetry code: (vii) −*x*+1, −*y*, −*z*+1.

### (II) Poly[µ-aqua-bis[µ3-(3,5-dichlorophenoxy)acetato]dirubidium] Crystal data

**Table:**

[Rb_2_(C_8_H_5_Cl_2_O_3_)_2_(H_2_O)]	*F*(000) = 1224
*M**_r_* = 629.00	*D*_x_ = 1.913 Mg m^−^^3^
Monoclinic, *C*2/*c*	Mo *K*α radiation, λ = 0.71073 Å
Hall symbol: -C 2yc	Cell parameters from 2435 reflections
*a* = 39.641 (3) Å	θ = 3.6–28.3°
*b* = 4.3302 (3) Å	µ = 5.01 mm^−^^1^
*c* = 12.8607 (8) Å	*T* = 200 K
β = 98.404 (5)°	Prism, colourless
*V* = 2183.9 (3) Å^3^	0.40 × 0.12 × 0.04 mm
*Z* = 4	

### (II) Poly[µ-aqua-bis[µ3-(3,5-dichlorophenoxy)acetato]dirubidium] Data collection

**Table:**

Oxford Diffraction Gemini-S CCD-detector diffractometer	2152 independent reflections
Radiation source: Enhance (Mo) X-ray source	1910 reflections with *I* > 2σ(*I*)
Graphite monochromator	*R*_int_ = 0.055
Detector resolution: 16.077 pixels mm^-1^	θ_max_ = 26.0°, θ_min_ = 3.2°
ω–scans	*h* = −45→48
Absorption correction: multi-scan (*CrysAlis PRO*; Agilent, 2013)	*k* = −5→5
*T*_min_ = 0.369, *T*_max_ = 0.980	*l* = −15→15
7520 measured reflections	

### (II) Poly[µ-aqua-bis[µ3-(3,5-dichlorophenoxy)acetato]dirubidium] Refinement

**Table:**

Refinement on *F*^2^	Primary atom site location: structure-invariant direct methods
Least-squares matrix: full	Secondary atom site location: difference Fourier map
*R*[*F*^2^ > 2σ(*F*^2^)] = 0.040	Hydrogen site location: inferred from neighbouring sites
*wR*(*F*^2^) = 0.095	H atoms treated by a mixture of independent and constrained refinement
*S* = 1.06	*w* = 1/[σ^2^(*F*_o_^2^) + (0.0491*P*)^2^] where *P* = (*F*_o_^2^ + 2*F*_c_^2^)/3
2152 reflections	(Δ/σ)_max_ = 0.003
136 parameters	Δρ_max_ = 0.98 e Å^−^^3^
1 restraint	Δρ_min_ = −1.00 e Å^−^^3^

### (II) Poly[µ-aqua-bis[µ3-(3,5-dichlorophenoxy)acetato]dirubidium] Special details

**Table:**

Geometry. Bond distances, angles etc. have been calculated using the rounded fractional coordinates. All su's are estimated from the variances of the (full) variance-covariance matrix. The cell esds are taken into account in the estimation of distances, angles and torsion angles
Refinement. Refinement of F^2^ against ALL reflections. The weighted R-factor wR and goodness of fit S are based on F^2^, conventional R-factors R are based on F, with F set to zero for negative F^2^. The threshold expression of F^2^ > 2sigma(F^2^) is used only for calculating R-factors(gt) etc. and is not relevant to the choice of reflections for refinement. R-factors based on F^2^ are statistically about twice as large as those based on F, and R- factors based on ALL data will be even larger.

### (II) Poly[µ-aqua-bis[µ3-(3,5-dichlorophenoxy)acetato]dirubidium] Fractional atomic coordinates and isotropic or equivalent isotropic displacement parameters (Å^2^)

**Table:**

	*x*	*y*	*z*	*U*_iso_*/*U*_eq_	
Rb1	0.53252 (1)	0.71425 (8)	0.41106 (2)	0.0271 (1)	
Cl3	0.66575 (3)	1.1071 (2)	0.31320 (7)	0.0394 (3)	
Cl5	0.72802 (2)	0.4700 (3)	0.64713 (9)	0.0510 (4)	
O1W	0.50000	0.2897 (8)	0.25000	0.0336 (12)	
O11	0.59805 (6)	0.4938 (6)	0.54449 (18)	0.0312 (8)	
O13	0.53789 (6)	0.2205 (5)	0.5570 (2)	0.0295 (8)	
O14	0.55505 (6)	0.0734 (6)	0.72371 (19)	0.0341 (8)	
C1	0.63017 (8)	0.5832 (8)	0.5286 (3)	0.0255 (11)	
C2	0.63168 (10)	0.7780 (8)	0.4420 (3)	0.0278 (11)	
C3	0.66324 (10)	0.8701 (8)	0.4215 (3)	0.0284 (11)	
C4	0.69371 (11)	0.7828 (8)	0.4829 (3)	0.0327 (12)	
C5	0.69102 (9)	0.5914 (9)	0.5678 (3)	0.0302 (11)	
C6	0.66010 (8)	0.4923 (8)	0.5924 (3)	0.0267 (11)	
C12	0.59553 (9)	0.3198 (8)	0.6376 (3)	0.0285 (11)	
C13	0.55928 (9)	0.1991 (8)	0.6381 (3)	0.0243 (11)	
H1W	0.4832 (8)	0.172 (8)	0.266 (4)	0.0510*	
H2	0.61150	0.84410	0.39880	0.0330*	
H4	0.71520	0.85090	0.46730	0.0390*	
H6	0.65920	0.36420	0.65190	0.0320*	
H121	0.60210	0.45220	0.70000	0.0340*	
H122	0.61160	0.14340	0.64200	0.0340*	

### (II) Poly[µ-aqua-bis[µ3-(3,5-dichlorophenoxy)acetato]dirubidium] Atomic displacement parameters (Å^2^)

**Table:**

	*U*^11^	*U*^22^	*U*^33^	*U*^12^	*U*^13^	*U*^23^
Rb1	0.0270 (2)	0.0340 (2)	0.0204 (2)	0.0005 (1)	0.0035 (2)	0.0014 (1)
Cl3	0.0502 (6)	0.0428 (6)	0.0275 (5)	−0.0119 (5)	0.0132 (5)	0.0037 (4)
Cl5	0.0231 (5)	0.0802 (8)	0.0474 (6)	−0.0029 (5)	−0.0022 (5)	0.0124 (6)
O1W	0.028 (2)	0.034 (2)	0.039 (2)	0.0000	0.0057 (19)	0.0000
O11	0.0205 (13)	0.0506 (16)	0.0227 (13)	−0.0044 (11)	0.0038 (11)	0.0129 (12)
O13	0.0245 (14)	0.0378 (14)	0.0255 (14)	−0.0029 (10)	0.0013 (12)	−0.0011 (11)
O14	0.0317 (14)	0.0491 (16)	0.0232 (13)	−0.0085 (13)	0.0100 (12)	0.0059 (12)
C1	0.0249 (19)	0.0317 (19)	0.0205 (18)	−0.0028 (16)	0.0051 (16)	−0.0045 (16)
C2	0.027 (2)	0.035 (2)	0.0215 (19)	−0.0002 (15)	0.0038 (17)	−0.0013 (15)
C3	0.037 (2)	0.0300 (19)	0.0194 (18)	−0.0075 (17)	0.0084 (17)	−0.0052 (15)
C4	0.028 (2)	0.044 (2)	0.028 (2)	−0.0104 (17)	0.0106 (18)	−0.0055 (17)
C5	0.0238 (19)	0.042 (2)	0.0241 (18)	−0.0042 (17)	0.0013 (16)	−0.0036 (17)
C6	0.0244 (19)	0.035 (2)	0.0207 (18)	−0.0020 (15)	0.0036 (15)	−0.0013 (16)
C12	0.025 (2)	0.040 (2)	0.0200 (18)	−0.0040 (16)	0.0018 (16)	0.0041 (16)
C13	0.024 (2)	0.0269 (18)	0.0231 (19)	0.0007 (15)	0.0071 (17)	−0.0048 (15)

### (II) Poly[µ-aqua-bis[µ3-(3,5-dichlorophenoxy)acetato]dirubidium] Geometric parameters (Å, º)

**Table:**

Rb1—O1W	2.924 (2)	O1W—H1W^iv^	0.89 (3)
Rb1—O11	3.050 (2)	C1—C6	1.397 (5)
Rb1—O13	2.832 (2)	C1—C2	1.405 (5)
Rb1—O13^i^	2.874 (2)	C2—C3	1.375 (6)
Rb1—O13^ii^	2.894 (2)	C3—C4	1.395 (6)
Rb1—O14^iii^	2.842 (2)	C4—C5	1.387 (5)
Cl3—C3	1.745 (4)	C5—C6	1.378 (5)
Cl5—C5	1.741 (4)	C12—C13	1.530 (5)
O11—C1	1.374 (4)	C2—H2	0.9500
O11—C12	1.431 (4)	C4—H4	0.9500
O13—C13	1.248 (5)	C6—H6	0.9500
O14—C13	1.261 (4)	C12—H121	0.9900
O1W—H1W	0.89 (3)	C12—H122	0.9900
			
O1W—Rb1—O11	116.93 (7)	H1W—O1W—H1W^iv^	110 (3)
O1W—Rb1—O13	88.71 (7)	Rb1^iv^—O1W—H1W^iv^	118 (3)
O1W—Rb1—O13^i^	157.69 (6)	O11—C1—C2	115.8 (3)
O1W—Rb1—O13^ii^	80.06 (5)	O11—C1—C6	124.0 (3)
O1W—Rb1—O14^iii^	76.32 (6)	C2—C1—C6	120.3 (3)
O11—Rb1—O13	54.24 (7)	C1—C2—C3	118.1 (3)
O11—Rb1—O13^i^	84.01 (7)	C2—C3—C4	123.3 (4)
O11—Rb1—O13^ii^	135.28 (7)	Cl3—C3—C4	117.8 (3)
O11—Rb1—O14^iii^	103.36 (7)	Cl3—C3—C2	118.9 (3)
O13—Rb1—O13^i^	98.73 (7)	C3—C4—C5	116.6 (4)
O13—Rb1—O13^ii^	87.72 (7)	Cl5—C5—C4	119.1 (3)
O13—Rb1—O14^iii^	143.47 (7)	C4—C5—C6	122.7 (4)
O13^i^—Rb1—O13^ii^	79.26 (7)	Cl5—C5—C6	118.2 (3)
O13^i^—Rb1—O14^iii^	107.73 (7)	C1—C6—C5	119.0 (3)
O13^ii^—Rb1—O14^iii^	121.19 (7)	O11—C12—C13	111.3 (3)
Rb1—O1W—Rb1^iv^	102.10 (11)	O13—C13—C12	119.7 (3)
Rb1—O11—C1	124.0 (2)	O14—C13—C12	113.3 (3)
Rb1—O11—C12	118.55 (19)	O13—C13—O14	126.9 (3)
C1—O11—C12	116.9 (3)	C1—C2—H2	121.00
Rb1—O13—C13	125.9 (2)	C3—C2—H2	121.00
Rb1—O13—Rb1^v^	98.73 (8)	C3—C4—H4	122.00
Rb1—O13—Rb1^ii^	92.28 (7)	C5—C4—H4	122.00
Rb1^v^—O13—C13	117.8 (2)	C1—C6—H6	121.00
Rb1^ii^—O13—C13	116.1 (2)	C5—C6—H6	120.00
Rb1^v^—O13—Rb1^ii^	100.74 (7)	O11—C12—H121	109.00
Rb1^vi^—O14—C13	134.3 (2)	O11—C12—H122	109.00
Rb1^iv^—O1W—H1W	105 (3)	C13—C12—H121	109.00
Rb1—O1W—H1W	118 (3)	C13—C12—H122	109.00
Rb1—O1W—H1W^iv^	105 (3)	H121—C12—H122	108.00
			
O11—Rb1—O1W—Rb1^iv^	−149.55 (5)	O13—Rb1—O13^ii^—Rb1^ii^	0.00 (7)
O13—Rb1—O1W—Rb1^iv^	162.30 (5)	O13—Rb1—O13^ii^—C13^ii^	−132.3 (2)
O1W—Rb1—O11—C1	101.0 (2)	O11—Rb1—O14^iii^—C13^iii^	88.7 (3)
O1W—Rb1—O11—C12	−87.7 (2)	O13—Rb1—O14^iii^—C13^iii^	42.2 (4)
O13—Rb1—O11—C1	167.6 (3)	Rb1—O11—C1—C2	−2.7 (4)
O13—Rb1—O11—C12	−21.0 (2)	Rb1—O11—C1—C6	177.2 (3)
O13^i^—Rb1—O11—C1	−87.1 (2)	C12—O11—C1—C2	−174.3 (3)
O13^i^—Rb1—O11—C12	84.3 (2)	C12—O11—C1—C6	5.7 (5)
O13^ii^—Rb1—O11—C1	−155.3 (2)	Rb1—O11—C12—C13	15.6 (3)
O13^ii^—Rb1—O11—C12	16.1 (3)	C1—O11—C12—C13	−172.4 (3)
O14^iii^—Rb1—O11—C1	19.7 (3)	Rb1—O13—C13—O14	147.4 (3)
O14^iii^—Rb1—O11—C12	−168.9 (2)	Rb1—O13—C13—C12	−35.8 (4)
O1W—Rb1—O13—C13	155.0 (3)	Rb1^v^—O13—C13—O14	−86.3 (4)
O1W—Rb1—O13—Rb1^v^	21.13 (6)	Rb1^v^—O13—C13—C12	90.5 (3)
O1W—Rb1—O13—Rb1^ii^	−80.10 (5)	Rb1^ii^—O13—C13—O14	33.2 (4)
O11—Rb1—O13—C13	29.9 (3)	Rb1^ii^—O13—C13—C12	−150.0 (2)
O11—Rb1—O13—Rb1^v^	−103.93 (9)	Rb1^vi^—O14—C13—O13	−90.5 (4)
O11—Rb1—O13—Rb1^ii^	154.83 (10)	Rb1^vi^—O14—C13—C12	92.5 (3)
O13^i^—Rb1—O13—C13	−46.2 (3)	O11—C1—C2—C3	−178.9 (3)
O13^i^—Rb1—O13—Rb1^v^	179.98 (11)	C6—C1—C2—C3	1.1 (5)
O13^i^—Rb1—O13—Rb1^ii^	78.77 (7)	O11—C1—C6—C5	178.8 (3)
O13^ii^—Rb1—O13—C13	−124.9 (3)	C2—C1—C6—C5	−1.3 (5)
O13^ii^—Rb1—O13—Rb1^v^	101.23 (7)	C1—C2—C3—Cl3	179.0 (3)
O13^ii^—Rb1—O13—Rb1^ii^	0.00 (7)	C1—C2—C3—C4	−0.7 (6)
O14^iii^—Rb1—O13—C13	90.3 (3)	Cl3—C3—C4—C5	−179.3 (3)
O14^iii^—Rb1—O13—Rb1^v^	−43.54 (14)	C2—C3—C4—C5	0.5 (5)
O14^iii^—Rb1—O13—Rb1^ii^	−144.77 (9)	C3—C4—C5—Cl5	179.4 (3)
O11—Rb1—O13^i^—Rb1^i^	127.64 (8)	C3—C4—C5—C6	−0.7 (6)
O11—Rb1—O13^i^—C13^i^	−11.0 (2)	Cl5—C5—C6—C1	−178.9 (3)
O13—Rb1—O13^i^—Rb1^i^	179.98 (10)	C4—C5—C6—C1	1.1 (6)
O13—Rb1—O13^i^—C13^i^	41.3 (2)	O11—C12—C13—O13	10.0 (4)
O11—Rb1—O13^ii^—Rb1^ii^	−29.37 (12)	O11—C12—C13—O14	−172.7 (3)
O11—Rb1—O13^ii^—C13^ii^	−161.6 (2)		

Symmetry codes: (i) *x*, *y*+1, *z*; (ii) −*x*+1, −*y*+1, −*z*+1; (iii) *x*, −*y*+1, *z*−1/2; (iv) −*x*+1, *y*, −*z*+1/2; (v) *x*, *y*−1, *z*; (vi) *x*, −*y*+1, *z*+1/2.

### (II) Poly[µ-aqua-bis[µ3-(3,5-dichlorophenoxy)acetato]dirubidium] Hydrogen-bond geometry (Å, º)

**Table:**

*D*—H···*A*	*D*—H	H···*A*	*D*···*A*	*D*—H···*A*
O1*W*—H1*W*···O14^vii^	0.89 (3)	1.87 (3)	2.750 (3)	171 (5)

Symmetry code: (vii) −*x*+1, −*y*, −*z*+1.
